# Utility of Serum Ki-67 as a Marker for Malignancy in Dogs

**DOI:** 10.3390/ani12101263

**Published:** 2022-05-14

**Authors:** Annkathrin Estaller, Martin Kessler, Axel Wehrend, Johannes Hirschberger, Stephan Neumann

**Affiliations:** 1 Small Animal Clinic, Institute of Veterinary Medicine, Georg-August University of Göttingen, 37077 Göttingen, Germany; a.estaller@googlemail.com; 2 Small Animal Clinic Hofheim, 65719 Hofheim am Taunus, Germany; m.kessler@tierklinik-hofheim.de; 3 Clinic of Obstetrics, Gynaecology and Andrology of Large and Small Animals of the Justus-Liebig-University Giessen, 35392 Giessen, Germany; axel.wehrend@vetmed.uni-giessen.de; 4 Clinic of Small Animal Medicine, Ludwig Maximilian University of Munich, 80539 Munich, Germany; j.hirschberger@medizinische-kleintierklinik.de

**Keywords:** biomarker, cancer immunology, dog, Ki-67, malignancy, serum, veterinary oncology

## Abstract

**Simple Summary:**

Although serum tumour markers offer an uncomplicated, non-invasive examination method and possible therapeutic options, they are still rarely used in veterinary medicine. Our marker of interest, the Ki-67 protein, can only be detected in the active phases of the cell cycle. Therefore, it is a suitable marker for assessing the proliferating cell fraction of an organism and can thus provide information about potentially present, rapid-growing tumour tissue. The purpose of our study was to determine whether Ki-67 could be considered as a possible tumour marker in canine serum for veterinary medicine. We measured serum concentrations of Ki-67 in dogs with various malignant tumours, such as carcinomas, sarcomas, and lymphomas. In the dogs with malignant tumours we determined significantly higher serum Ki-67 concentrations compared with healthy dogs and dogs with non-malignant diseases. No significant difference in serum Ki-67 concentration was observed between the different types of cancer or between benign and malignant mammary tumours. Our investigations also included some inflammatory parameters measured in blood, such as neutrophils, lymphocytes, and monocytes, with mixed results. The results of our study suggest that Ki-67 may be useful as a potential serum tumour marker, providing information about the presence of malignant diseases in a dog.

**Abstract:**

Tumour markers are scarcely used in veterinary medicine, although they are non-invasive, contribute to a faster diagnosis and new therapeutic options. The nuclear protein Ki-67 is absent in G_0_-phase but is detectable throughout all active phases of the cell cycle. Consequently, it is used as a marker for the proliferating cell fraction of a cell population and thus could indicate neoplastic tissue present. Our study is designed to show whether Ki-67 can be considered as a potential canine serum tumour marker for veterinary medicine. We measured serum concentrations of Ki-67 in dogs with various malignant tumours (carcinomas (*n* = 35); sarcomas (*n* = 26); lymphomas (*n* = 21)) using a commercially available quantitative sandwich ELISA from mybiosource. Dogs with malignant tumours showed significantly higher serum Ki-67 concentrations compared to healthy dogs (*n* = 19) and non-neoplastic diseased dogs (*n* = 26). No significant difference in serum Ki-67 concentration was detected between carcinoma, sarcoma, and lymphoma, nor between mammary adenocarcinoma and adenoma. In our investigations we also included some inflammatory parameters measured in blood, such as neutrophils, lymphocytes, and monocytes, and gained mixed results. The results of our study suggest that Ki-67 may be useful as a potential serum tumour marker, providing information about the presence of malignancies in a dog.

## 1. Introduction

The identification of tumour markers is a valuable investigative method in oncology. It differs from other investigative methods, such as imaging and histology, as it is less invasive, non-complex, and inexpensive to perform [1,2]. In humans, numerous tumour markers have been described that are indicative for the potential presence of tumour tissue, the course and prognosis of the disease, and the detection of recurrences [2,3]. In veterinary medicine, only a few tumour markers are used in clinical practice so far [4,5]. It has been shown that their diagnostic utility is limited due to low sensitivity and specificity [5,6]. Malignant tumour tissue differs from other tissues particularly due to its higher proliferation rate [7,8].

Therefore, molecules that are increasingly expressed in the context of cell proliferation are very appealing for their use as tumour markers.

A promising biomarker strictly associated with cell proliferation is the nuclear Ki-67 protein, found in 1983 in a Hodgkin lymphoma cell line [9,10]. The function of the Ki-67 protein is not yet fully understood [11]. Functioning as a surfactant, it stabilizes the chromosomes after the dismantling of the nuclear membrane during mitosis and thus ensures smooth separation of the chromosomes and successful interaction with the mitotic spindle [12].

As a proliferation marker, Ki-67 protein is absent in resting cells, but can be identified during all active phases of the cell cycle. Throughout the interphase it can solely be detected within the nucleus, although in mitosis the majority of the Ki-67 antigen is relocated onto the surface of the chromosomes [10,11].

During interphase and mitosis, Ki-67 is also found to some extent in the domain of rRNA transcription, where it is considered to play an important role, one reason being that its removal inhibits ribosomal RNA synthesis [11,13,14].

Its selective expression in active phases during the cell cycle makes the Ki-67 protein an interesting target in cancer research. Ki-67 is already widely used in immunohistochemistry to determine the growth fraction of the cell population in question [15,16]. The percentage of Ki-67-positive tumour cells is often correlated with therapeutic success and prognosis of patients suffering from cancer.

Another contributor to tumour growth and progression are inflammatory cells, for example neutrophils, lymphocytes, and monocytes play an important role in the cancer microenvironment of tumour tissue or behave differently in it compared to a healthy organism [17,18,19,20].

Consequently, the relationship between tumour markers, such as Ki-67, and inflammatory cells are increasingly becoming the focus of some research approaches to discover possible correlations and improve tumour diagnostics [21,22].

Ki-67 in itself has been investigated in humans in a variety of malignancies, especially prostate and breast carcinomas, brain tumours, nephroblastoma and neuroendocrine neoplasms [16,23,24,25,26].

There are significantly less studies investigating Ki-67 as a serum tumour marker, it has been shown however, that women with malignant breast cancer have higher serum Ki-67 levels compared to control groups with non-malignant diseases [27,28].

To the authors’ knowledge there are no studies investigating Ki-67 in serum as cancer biomarker in veterinary medicine. In a pilot study, our group evaluated serum Ki-67 levels in a small cohort of 20 dogs with malignant tumours and compared them to serum Ki-67 levels in clinically healthy dogs and dogs with non-malignant diseases [29].

Based on the promising results of this pilot study the aim of the present retrospective study was to investigate serum Ki-67 in larger groups of dogs with malignant tumours, to detect differences in regard to tumour entity, whether there is a correlation between Ki-67 and certain inflammatory cells measured in the blood and whether serum Ki-67 could be a potential pan tumour marker for future screening tests for canine malignancies.

## 2. Materials and Methods

### 2.1. Animals

A total of 140 dogs were examined and classified into four different groups dependent on their health status: healthy, non-neoplastic diseased, benign tumours (mammary adenoma), and malignant tumours (mammary adenocarcinoma, squamous cell carcinoma of the oral and nasal mucosa and the dermis, soft-tissue sarcoma, such as subcutaneous spindle cell sarcoma, oral fibrosarcoma and splenic hemangiosarcoma, and multicentric, cutaneous and gastrointestinal lymphoma). All dogs were diagnosed between January 2018 and December 2019.

The dogs in the healthy control group (*n* = 19) had been brought to the University of Göttingen’s veterinary clinic for small animals (Göttingen, Germany) for a general examination. This group of dogs had to be free of any indications of disease for at least two months prior to the clinical assessment and for four weeks thereafter. A complete blood count was performed on the respective patient and all the blood parameters had to be within the laboratory reference interval to be considered for the control group.

The experimental procedures for blood sampling in healthy dogs were approved by the regional authorities for Consumer Protection and Food Safety Acts in Niedersachsen, Germany (reference n° 33.9-42502-05-17A148).

The non-neoplastic diseased dogs (*n* = 26) were brought to the veterinary clinic for small animals at the University of Göttingen (Göttingen, Germany) due to various illnesses. A clinical examination, diagnostic imaging (thoracic radiography and abdominal ultrasonography), and lab work were performed on all dogs. The dogs in this group were each affected by a non-neoplastic systemic disease, such as diabetes, canine idiopathic epilepsy, chronic renal or heart failure, gastroenteritis, endometritis, urinary tract diseases, hepatitis, food poisoning, or a cervical abscess.

The owners admitted the dogs of the tumour groups to the veterinary clinic for small animals at the University of Göttingen (Göttingen, Germany), the oncology service of the veterinary clinic for small animals in Hofheim (Hofheim, Germany), or the veterinary clinic for gynaecology and obstetrics at the University of Gießen (Gießen, Germany) due to the symptoms brought on by their particular tumour diseases. To confirm their cancer diagnoses, those dogs were subjected to all the necessary diagnostic procedures.

Specialized veterinary pathologists generated histopathologic diagnoses utilizing incisional or excisional biopsy specimens of the tumours in question. Tumour volume was determined by multiplying width by height and by depth, except for lymphoma, since it is a systemic disease.

The malignant tumour group consisted of 82 dogs with different malignant tumour diseases (carcinomas *n* = 35; soft tissue sarcomas *n* = 26; malignant lymphomas *n* = 21).

A minimum follow-up time of 2 years following the cancer diagnosis was documented for all dogs with malignant tumours. Cause of death was recorded as tumour-related or non-tumour related in all cases that died during the observation period.

The group of dogs with histopathologically confirmed benign masses consisted of 13 dogs with mammary adenomas. Other diseases, especially malignant tumour diseases, were ruled out in these cases by multiple follow-up examinations.

The group of dogs with mammary adenomas was compared to the group of dogs with mammary adenocarcinomas.

### 2.2. Histopathology

Histology was performed using routinely embedded paraffin sections after hematoxylin and eosin staining. The tumour entity, as well as the histologic grade was evaluated. Diagnoses were made by veterinary pathologists according to the WHO guidelines [30].

In case of mammary tumours in particular, malignancy was graded according to the classification system established by Peña et al. 2012 [31].

### 2.3. Blood Sample Collection and Processing

Blood samples used for all measurements were collected from the cephalic veins prior to any treatments. Blood samples for complete blood counts were collected in polypropylene tubes containing 1.6 mg ethylenediamine tetraacetic acid/mL blood (Fa. Sarstedt AG & Co., Nümbrecht, Germany) and analysed using a ProCyte Dx Hematology Analyzer (IDEXX Laboratories, Inc., Westbrook, ME, USA), which counted red blood cells, white blood cells, haemoglobin and platelets, among other things. Some of these values were also used for subsequent analyses related to our measured serum Ki-67 concentrations, which are discussed in more detail below.

Standard serum tubes (Fa. Sarstedt AG & Co., Nümbrecht, Germany) were used to take serum samples for serum chemistry and then centrifuged in an Eppendorf centrifuge 5424 (Fa. Eppendorf AG, Hamburg, Germany) at 3000× *g* for 5 min. The serum was then removed from the tube and analysed using the clinical chemistry analyser (Konelab 20i; Fa. Thermo Fischer Scientific Inc., Dreieich, Germany) and commercial kits according to standard procedures.

The serum for Ki-67 measurements was allowed to rest at room temperature for 2 h and then centrifuged at 1000× *g* for 15 min. It was then pipetted into Eppendorf tubes, frozen at −80 °C and stored under these conditions until processed within the next 3 months.

### 2.4. Immunoassay

The Ki-67 concentrations were measured using a commercially available immunoassay kit [Canine Ki-67 Protein (Ki67P) ELISA kit; MyBioSource, San Diego, CA, USA (Cat.No: MBS089640), quantitative Sandwich ELISA] with non-diluted serum in duplicate, adhering to the manufacturer’s instructions.

First, serum samples and standards were pipetted into the appropriate wells of the microtiter plate. HRP conjugate reagent was then added and the microtiter plate was incubated at 37 °C for 1 h. The Ki-67 protein present in the samples bound to the immobilized Ki-67 antibodies on the microtiter plate, and at the same time, the detection antibody against Ki-67 contained in the HRP conjugate reagent in turn bound to the Ki-67 antigens from the samples. To remove all unbound antibodies, four manual washing steps were then performed. Subsequently, an enzymatic reaction was induced by adding chromogen solution and incubating again at 37 °C for 15 min, which became visible in the form of a blue colour complex. The resulting colour intensity is directly proportional to the amount of Ki-67 protein bound. In the final step, a stop solution was added to terminate the enzyme-substrate reaction, resulting in a colour change to yellow. The optical density (O.D.) of each well was then measured spectrophotometrically at a wavelength of 450 nm. Ki-67 concentrations in ng/mL were then calculated using the standard curve generated by the CurveExpert Professional program (Hyams Development, https://www.curveexpert.net/ (accessed on 17 May 2018)). According to the manufacturer, the detection range of the ELISA in serum samples is 0.625–20.0 ng/mL. The mean intra- and inter-assay coefficients are reported to be less than 15% each [32].

### 2.5. Ratios

To distinguish between the group of benign mammary adenomas and the malignant mammary adenocarcinomas, ratios were formed in order to compare those two groups.

Ratios were calculated by dividing the serum Ki-67 concentrations by the neutrophil granulocyte, the lymphocyte and the monocyte counts, respectively. Similarly, for completeness, blood concentrations of neutrophil granulocytes, lymphocytes, and monocytes of all the various groups were compared with each other. As mentioned above, apart from the Ki-67 concentrations measured by ELISA, the cell counts used were obtained from the accompanying routine blood count.

### 2.6. Statistical Analysis

Statistical examination was performed using the program Prism 9 (GraphPad Software, San Diego, CA, USA). Data were tested for normal distribution using the Shapiro–Wilk and the Kolmogorov–Smirnov tests, the Pearson’s correlation coefficient was used to test for linear correlation between the datasets. Non-normally distributed data were compared using the Mann–Whitney (two groups) or the Kruskal–Wallis (multiple groups) tests. Linear regression analyses were performed to examine dependency of Ki-67 on tumour volume. Receiver operation characteristic (ROC) curves were calculated for discriminating healthy dogs from tumour patients. A *p*-value < 0.05 was considered statistically significant.

## 3. Results

A wide variety of dog breeds and mixed breeds were represented and no dog breed was predominant (Table 1). Information on age and sex distribution in the different groups is summarized in Table 2.

**Figure 1:**
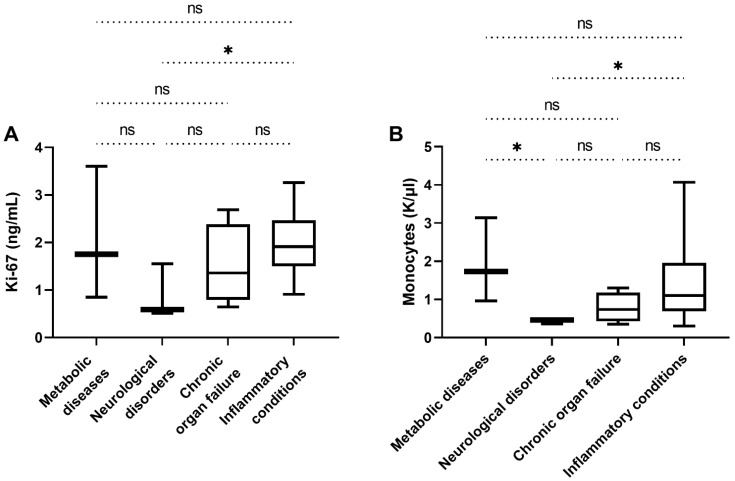
Serum Ki-67 concentrations and monocyte blood counts within the group of dogs with non-neoplastic diseases. (**A**) Comparing the Ki-67 levels, no significant difference between dogs with metabolic disorders, neurological disorders, inflammatory conditions, or organ failure could be found (*p* = 0.11), apart from significantly higher Ki-67 concentration in dogs with inflammatory diseases in comparison to dogs with neurological disorders (*p* = 0.02). (**B**) Monocyte blood counts were significantly lower in the neurologic disorder subgroup compared with the metabolic and inflammatory disease subgroups (*p* = 0.025 and *p* = 0.03). There was no significant difference between the other subgroups of the non-neoplastic diseased dogs. (ns) not significant; (*) *p* < 0.05.

**Figure 2:**
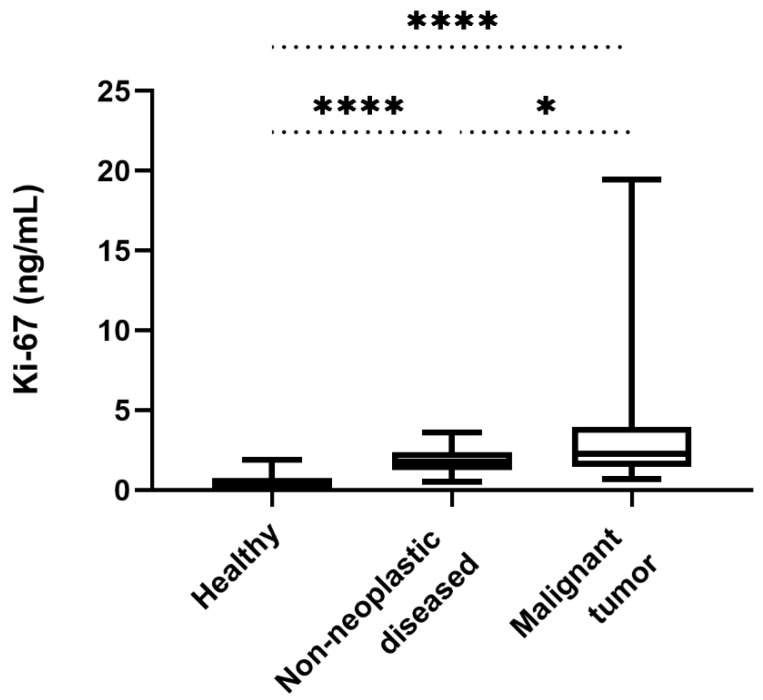
Comparison of the serum Ki-67 concentrations in healthy dogs, dogs with non-neoplastic diseases, and dogs with malignant tumours. Concentrations between healthy dogs and those suffering from non-neoplastic diseases differed significantly (*p* < 0.0001). The dogs with malignant tumours had significantly higher Ki-67 concentrations than healthy dogs (*p* < 0.0001) and those with non-neoplastic diseases (*p* = 0.027). (*) *p* < 0.05; (****) *p* < 0.0001.

The healthy control group (*n* = 19) had serum Ki-67 concentrations between 0 and 1.88 ng/mL (median of 0.29 ng/mL). The dogs in the group with non-neoplastic diseases (*n* = 26) suffered from metabolic diseases (diabetes *n* = 2; hypoadrenocorticism *n* = 1), neurological disorders (canine idiopathic epilepsy *n* = 2; geriatric vestibular syndrome *n* = 1), chronic organ failure (chronic renal failure *n* = 3; chronic heart failure *n* = 1) and inflammatory conditions (gastroenteritis *n* = 6; endometritis *n* = 3; other inflammatory diseases *n* = 7). In this group Ki-67 concentrations ranged from 0.51 to 3.6 ng/mL (median 1.79 ng/mL). There was no significant difference between Ki-67 levels in dogs with metabolic disorders, neurological disorders, inflammatory conditions, or organ failure (*p* = 0.11), with the exception of a significantly higher Ki-67 concentration in dogs with inflammatory diseases compared to dogs with neurological disorders (*p* = 0.02, Figure 1A). There was a highly significant difference in the serum Ki-67 concentrations between healthy dogs and dogs with non-neoplastic diseases (*p* < 0.0001, Figure 2).

The group of dogs with malignant tumours consisted of 82 dogs with different malignant tumour diseases that were subclassified into three groups. The carcinoma group included malignant subtypes of mammary tumours classified by the grading system established by Peña et al. 2012 (adenocarcinomas *n* = 26) and nine squamous cell carcinomas of the dermis (*n* = 4), oral mucosa (*n* = 3), and nasal mucosa (*n* = 2).

The mesenchymal tumour group (*n* = 26) included soft-tissue sarcomas of different anatomic locations, including subcutaneous spindle cell sarcomas (*n* = 17) and splenic hemangiosarcomas (*n* = 9). In the lymphoma group dogs with various subtypes of high-grade lymphomas were included (multicentric lymphoma (*n* = 18); cutaneous epitheliotropic lymphoma (*n* = 2), and gastrointestinal lymphoma (*n* = 1)).

Serum Ki-67 concentrations in dogs with malignant tumours ranged between 0.69 and 19.46 ng/mL (median 2.26 ng/mL).

**Figure 3:**
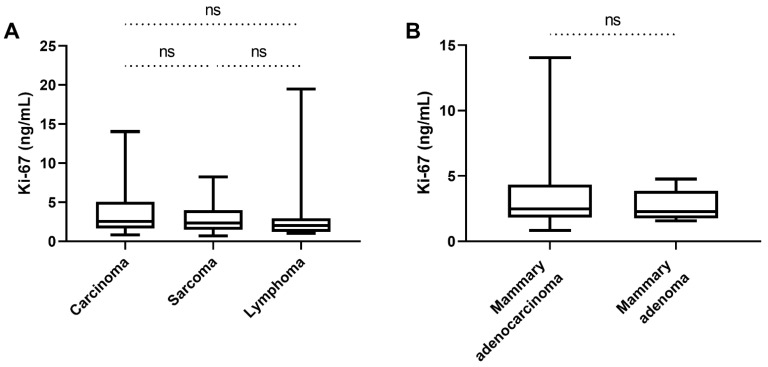
Serum Ki-67 concentrations within the group of dogs with tumour diseases. (**A**) Comparison of the serum Ki-67 concentrations of the different malignant tumour groups (epithelial tumours, mesenchymal tumours, and lymphoma). No significant difference could be found comparing these 3 groups of tumours (*p* = 0.37). (**B**) When comparing the serum Ki-67 concentrations of dogs with mammary adenocarcinoma with those of the dogs with mammary adenoma, the tumour serum Ki-67 concentrations did not differ significantly (*p* = 0.42). (ns) not significant.

In the carcinoma group, dogs with mammary gland adenocarcinomas (n = 26) had serum Ki-67 concentrations between 0.83 and 14.04 ng/mL (median 2.48 ng/mL). Dogs with squamous cell carcinomas (*n* = 9) had serum Ki-67 concentrations between 1.06 and 13.63 ng/mL (median 2.68 ng/mL). Dogs with mesenchymal tumours (*n* = 26) had serum Ki-67 concentrations between 0.69 and 8.27 ng/mL (median 2.35 ng/mL). Of these, subcutaneous spindle cell sarcoma (*n* = 17) and splenic hemangiosarcoma (*n* = 9) had serum Ki-67 levels between 0.69 and 8.27 ng/mL (median 2.42 ng/mL) and between 0.99 and 7.19 ng/mL (median 2.27 ng/mL), respectively. Ki-67 concentrations in dogs with high grade lymphomas (*n* = 21) were between 1.01 and 19.46 ng/mL (median 2.0 ng/mL). There were no statistically significant differences in Ki-67 levels between carcinoma, sarcoma, and lymphoma subgroups (*p* = 0.37; Figure 3A).

In the dogs with benign masses (mammary adenoma, *n* = 13) serum Ki-67 concentrations ranged between 1.55 and 4.75 ng/mL (median 2.28 ng/mL).

**Figure 4:**
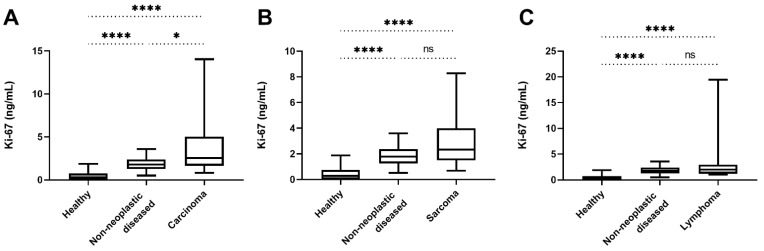
Serum Ki-67 concentrations of the different malignant tumour groups were also compared individually with the healthy group, and the non-neoplastic diseased group. (**A**) The Ki-67 concentrations of the carcinoma group differed significantly compared to the healthy control group (*p* < 0.0001) and compared to the group of dogs with non-neoplastic diseases (*p* = 0.0198). (**B**) A comparison of the Ki-67 concentrations of the sarcoma group revealed significantly higher concentrations than the ones in the healthy control group (*p* < 0.0001), whereas concentrations in the group with non-neoplastic diseases did not differ significantly compared to the sarcoma group (*p* = 0.102). (C) The Ki-67 concentrations of the lymphoma group were significantly higher than the ones of the healthy dogs (*p* < 0.0001), however, compared to the group with non-neoplastic diseases, there was no significant difference (*p* = 0.439). (ns) not significant; (*) *p* < 0.05; (****) *p* < 0.0001.

There was a significant difference in the Ki-67 serum concentrations of dogs with malignant tumours compared to healthy dogs and to dogs with non-neoplastic diseases (*p* < 0.0001 and *p* = 0.027, respectively; Figure 2). If the individual groups of the malignant tumour group are considered separately and compared with the healthy group and the non-neoplastic diseased group, significantly higher values were found in each case compared with the healthy group (*p* < 0.0001 in each case; Figure 4A–C). In comparison with the non-neoplastic diseased group, however, only the carcinoma group showed significantly higher values (*p* = 0.02; Figure 4A); there was no significant difference in the sarcoma group and the lymphoma group (*p* = 0.10 and *p* = 0.44, respectively; Figure 4A–C).

No significant differences in serum Ki-67 concentrations between mammary adenocarcinoma and mammary adenoma were present (*p* = 0.42; Figure 3B).

**Figure 5:**
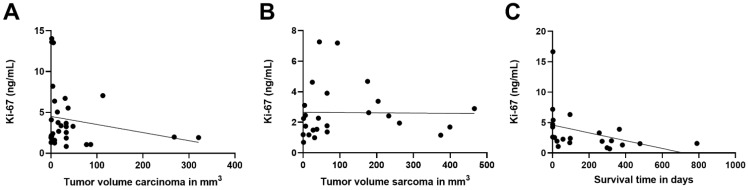
Linear regression analysis. (**A**) The influence of the tumour volume (mm^3^) on the serum Ki-67 concentrations was measured by linear regression analysis for carcinoma. No significant relationship could be found (*p* = 0.28; *r^2^* = 0.038). (**B**) Neither did the measured tumour volume (mm^3^) of the sarcomas affect their Ki-67 concentrations also measured by linear regression analysis (*p* = 0.955; *r^2^* = 0.0001). (**C**) The influence of the serum Ki-67 concentration on the survival time calculated with a linear regression analysis revealed no significant relation (*p* = 0.075; *r^2^* = 0.15).

The calculated tumour volumes of the tumours in the carcinoma group and in the sarcoma group are presented in Table 3. Tumour volume of neither carcinomas nor sarcomas showed a noticeable influence on serum Ki-67 concentrations when tested by linear regression (*p* = 0.28 and *p* = 0.955, respectively; Figure 5A,B).

Within the 2 years follow-up period, 22 of the patients with malignant tumours died as a result of their disease with survival times from 1 to 789 days (mean 168 days). No statistically significant correlation between serum Ki-67 concentrations and survival times was found (*p* = 0.075; Figure 5C).

**Figure 6:**
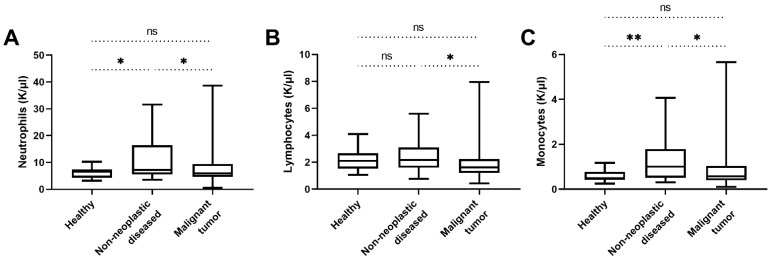
Comparison of different inflammatory parameters of healthy dogs, dogs with non-neoplastic diseases, and the different malignant tumour groups. (**A**) The neutrophil counts of the non-neoplastic diseased dogs were significantly higher than those of the healthy and the malignant tumour group (*p* = 0.031 and *p* = 0.023, respectively). The neutrophil counts between the healthy and the malignant tumour group did not differ significantly (*p* = 0.59). (**B**) The group of non-neoplastic diseased dogs had significantly higher lymphocyte counts than the malignant tumour group (*p* = 0.011). No significant difference between the lymphocyte counts of the healthy and the non-neoplastic diseased dogs and between the healthy and the malignant tumour group was found (*p* = 0.77 and *p* = 0.056, respectively). (**C**) The monocyte counts of the non-neoplastic diseased dogs were significantly higher than those of the healthy and the malignant tumour group (*p* = 0.006 and *p* = 0.011, respectively). The monocyte counts between the healthy and the malignant tumour group did not differ significantly (*p* = 0.34). ns, not significant. (*) *p* < 0.05; (**) *p* < 0.01.

The concentrations of inflammatory cells such as neutrophils, lymphocytes, and monocytes measured in the concomitant blood test are summarized in Table 4. When comparing the inflammation levels between the healthy control group, the non-neoplastic diseased group and the malignant tumour group, the following correlations were found. The non-neoplastic diseased group showed significantly higher concentrations of neutrophils than the healthy group and the malignant tumour group (*p* = 0.031 and *p* = 0.023, respectively; Figure 6A). Additionally, the non-neoplastic diseased group of dogs showed significantly higher lymphocyte counts compared to the malignant tumour group (*p* = 0.011; Figure 6B), the difference to the healthy control group is not significant here. The monocyte concentrations of the non-neoplastic diseased group were also significantly higher compared to both the healthy group and the malignant tumour group (*p* = 0.006 and *p* = 0.011, respectively; Figure 6C). Within the group of non-neoplastic diseased dogs, there was no significant difference in inflammatory parameters, with the exception of significantly lower monocyte counts in the neurologic disorder subgroup compared with the metabolic and inflammatory disease subgroups (*p* = 0.025 and *p* = 0.03, respectively; Figure 1B). If one compares the individual malignant tumour groups with one another, there is no significant difference in the aforementioned inflammatory parameters between the carcinoma group, the mesenchymal tumour group, and the lymphoma group. Looking specifically at the inflammatory parameters of malignant mammary adenocarcinomas compared with benign mammary adenomas, there is no statistical difference here either (*p* = 0.52, *p* = 0.39, and *p* = 0.43, respectively).

**Figure 7:**
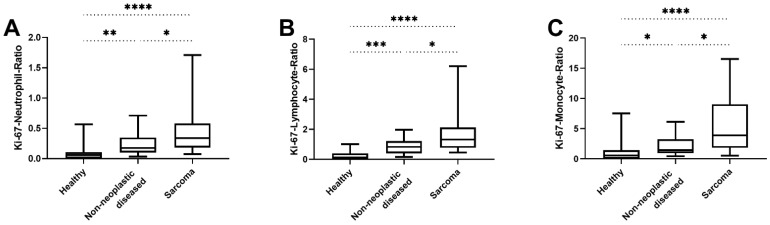
Comparison of ratios between Ki-67 and different inflammatory parameters of healthy dogs, dogs with non-neoplastic diseases, and the sarcoma group. (**A**) The Ki-67–neutrophil ratios of the non-neoplastic diseased group were significantly lower than those of the sarcoma group (*p* = 0.04). The healthy dogs also had significantly lower values than both the non-neoplastic diseased and the sarcoma group (*p* = 0.001 and *p* < 0.0001, respectively). (**B**) The Ki-67-lymphcyte-ratios of the non-neoplastic diseased group also were significantly lower than those of the sarcoma group (*p* = 0.03). The healthy dogs had significantly lower Ki-67–lymphocyte ratios than both the non-neoplastic diseased and the sarcoma group (*p* = 0.0002 and *p* < 0.0001, respectively). (**C**) The Ki-67–monocyte ratios of the non-neoplastic diseased group were significantly lower than those of the sarcoma group (*p* = 0.02). The healthy dogs also had significantly lower values than both the non-neoplastic diseased and the sarcoma group (*p* = 0.019 and *p* < 0.0001, respectively). (*) *p* < 0.05; (**) *p* < 0.01; (***) *p* < 0.001; (****) *p* < 0.0001.

**Figure 8:**
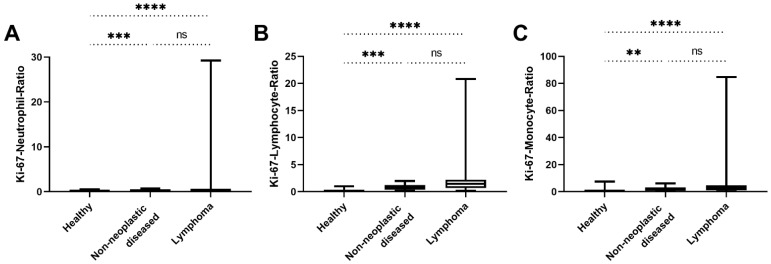
Comparison of ratios between Ki-67 and different inflammatory parameters of healthy dogs, dogs with non-neoplastic diseases, and the dogs with lymphoma. (**A**) The Ki-67–neutrophil ratios of the healthy dogs were significantly lower than those of the non-neoplastic diseased and the lymphoma group (*p* = 0.001 and *p* < 0.0001, respectively). The Ki-67–neutrophil ratios between the non-neoplastic diseased and the malignant tumour group did not differ significantly (*p* = 0.076). (**B**) The Ki-67–lymphocyte ratios of the healthy dogs were also significantly lower than those of the non-neoplastic diseased and the lymphoma group (*p* = 0.0002 and *p* < 0.0001, respectively). Additionally, the Ki-67–lymphocyte ratios between the non-neoplastic diseased and the lymphoma group did not differ significantly (*p* = 0.104). (**C**) The Ki-67-monocyte ratios of the non-neoplastic diseased dogs were significantly higher than those of the healthy group and the healthy group had significantly lower values than the lymphoma group (*p* = 0.005 and *p* < 0.0001, respectively). The Ki-67–monocyte ratios between the non-neoplastic diseased and the lymphoma group did not differ significantly (*p* = 0.16). (ns) not significant; (**) *p* < 0.01; (***) *p* < 0.001; (****) *p* < 0.0001.

Similarly, establishing ratios between serum Ki-67 concentrations and neutrophil, lymphocyte, and monocyte counts as indicators of inflammation, as well as components of the tumour microenvironment, was unable to distinguish mammary adenocarcinomas from adenomas (*p* = 0.83, *p* = 0.50 and *p* = 0.45, respectively; Table 5). However, comparing ratios of serum Ki-67 concentrations and neutrophils, lymphocytes, or monocytes of the sarcoma group with the non-neoplastic diseased group, a statistically significant difference was found in all three cases, with the diseased group showing lower values than the sarcoma group (*p* = 0.04, *p* = 0.03 and *p* = 0.02, respectively; Table 5; Figure 7A–C). This observation cannot be reproduced when comparing ratios of serum Ki-67 concentrations and neutrophils, lymphocytes, or monocytes of the lymphoma group with the non-neoplastic diseased group (*p* = 0.076, *p* = 0.104 and *p* = 0.16, respectively; Table 5; Figure 8A–C).

**Figure 9:**
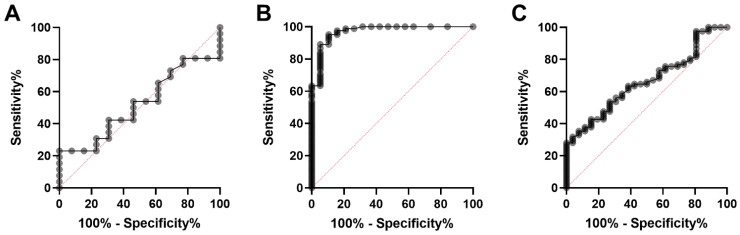
ROC curve analysis of serum Ki-67 concentration. (**A**) ROC curve for discriminating mammary adenocarcinoma from mammary adenoma. (**B**) ROC curve shown to distinguish healthy dogs from patient with tumour diseases. (**C**) ROC curve for discriminating non-malignant diseased dogs from tumour patients.

A receiver operating characteristic (ROC) curve was calculated to discriminate dogs with benign mammary adenoma from those with malignant mammary adenocarcinoma. The area under the curve (AUC) was 0.52 (*p* = 0.83; 95% confidence interval, 0.34–0.70). Using a cut-off value of 2.34 ng/mL, the sensitivity was 53.9% (confidence interval, 0.35–0.71) and the specificity 53.9% (confidence interval, 0.29–0.77; Figure 9A). Comparison of the ratios of Ki-67 to the inflammatory parameters neutrophils, lymphocytes, and monocytes of mammary adenoma and mammary adenocarcinoma did not result in a notable improvement in the AUC value. The area under the curve to discriminate healthy dogs from those with malignant tumours was 0.97 (*p* < 0.0001; 95% confidence interval, 0.93–1.0). Using a cut-off value of 1.15 ng/mL, the sensitivity was 89.0% (confidence interval, 0.80–0.94) and the specificity 94.7% (confidence interval, 0.75–0.99; Figure 9B). The ROC curve to separate non-neoplastic diseased dogs from the group of dogs with malignancies resulted in an AUC of 0.66 (*p* = 0.014; 95% confidence interval, 0.55–0.77). A cut-off value of 1.9 ng/mL resulted in a sensitivity of 63.4% (confidence interval, 0.53–0.73) and a specificity of 61.5% (confidence interval, 0.43–0.78; Figure 9C).

**Figure 10:**
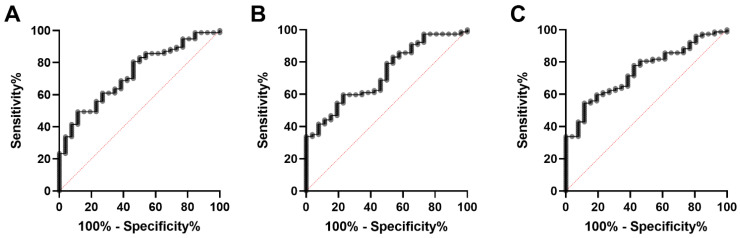
ROC curve analysis for discriminating non-neoplastic diseased dogs from tumour patients. (**A**) Ki-67-neutrophil ratio. (**B**) Ki-67-lymphocyte ratio. (**C**) Ki-67-monocyte ratio.

Using ratios of serum Ki-67 concentrations and the aforementioned inflammatory parameters for the ROC curve to distinguish non-neoplastic diseased dogs from tumour-diseased dogs, the AUC improves as follows: the comparison of the Ki-67–neutrophil ratio provides an AUC of 0.73 (*p* = 0.0005; 95% confidence interval, 0.63–0.83), with a sensitivity of 68.8% (confidence interval, 0.58–0.78) and a specificity of 61.5% (confidence interval, 0.43–0.78), when using a cut-off value of 0.24 (Figure 10A). Applying the Ki-67–lymphocyte ratio results in an AUC of 0.73 (*p* = 0.0006; 95% confidence interval, 0.62–0.83) and, with a cut-off of 1.05, a sensitivity of 61.0% (confidence interval, 0.50–0.71) and a specificity of 61.5% (confidence interval, 0.43–0.78; Figure 10B). The application of the Ki-67–monocyte ratio leads to an AUC of 0.74 (*p* = 0.0002; 95% confidence interval, 0.64–0.84). Using a cut-off value of 2.15, the sensitivity and specificity was 71.4% (confidence interval, 0.61–0.80) and 61.5% (confidence interval, 0.43–0.78), respectively (Figure 10C).

## 4. Discussion

Based on the WHO definition of biomarkers, a tumour marker is a measurable substance that provides information about the existence of tumour tissue in a body [1]. In order to allow for a good differentiation between healthy and tumour bearing dogs, tumour marker must be specific for tumour tissue.

Ki-67 is a specific biomarker for tumour proliferation, expressed in cells during the G phase of mitosis [11,33]. Many tumour tissues have a relatively high mitotic rate [34] and, therefore, show increased levels of Ki-67 that can be measured by immunohistochemistry [23]. Ki-67 immunohistochemistry is also frequently associated with the biologic behaviour of malignancies, as has been demonstrated, for example, in canine mast cell tumours [35].

In a pilot study investigating the expression of Ki-67 in cell cultures, to be precise cultured fibrosarcoma tumour cells, increased amounts of Ki-67 were found in culture supernatants during the proliferation phase of the cells [29]. In another study on canine mammary tumour cells, Ki-67 protein expression decreased after treatment with the tyrosine kinase inhibitor Masitinib mesylate [36].

Ki-67 concentrations were also evaluated in serum, showing comparable results, although a different immunoassay kit was used than in our study [29]. Further, a group of healthy dogs with nondetectable serum Ki-67 concentrations was compared to groups of dogs with different non-malignant diseases and with malignant tumours. Serum Ki-67 concentrations were between 0 and 69 pg/mL (median 0 pg/mL) and 0 and 7500 pg/mL (median 243 pg/mL), respectively, demonstrating a highly significant difference in the serum Ki-67 concentrations between all three groups [29]. The results of the current study are quite similar, although the serum Ki-67 concentrations differ slightly, probably due to the use of different ELISA kits. When establishing Ki-67 as a serum tumour marker, it is therefore recommended to always use the same ELISA kit or to work with individual cut-offs for the respective test kit used.

Based on these observations, we consider Ki-67 to be a valuable tumour marker indicating the presence of tumour tissue in the body. All 19 healthy patients in our study had serum Ki-67 concentrations in the lower detection range of the ELISA. Since there were no outliers in this particular group, we consider a Ki-67 serum concentration in the low picogram range to be biological for healthy dogs.

The very low baseline concentrations measurable in the serum of healthy dogs is to be expected, since as a proliferation marker, a certain level of Ki-67 will also be present from rapidly proliferating normal tissues in a healthy organism, e.g., from dividing normal mucosal or bone marrow cells [11].

In a study in human breast cancer patients, a baseline serum Ki-67 concentration was also present in the serum of the healthy control group. Similarly to our study, the level in tumour-free individuals was significantly lower than in the tumour group [37].

The control group of diseased dogs without malignancies in this study were affected by various inflammatory, metabolic, or degenerative processes. The serum Ki-67 concentrations in this group were also significantly lower than in the group of dogs with malignancies, but were significantly higher compared to the healthy dogs. This may be explained by a proliferation of immune cells in inflammatory processes [38]. Likewise, a higher expression of Ki-67 has been demonstrated in immune-mediated diseases in both humans and dogs [39,40].

Acute inflammatory diseases in the group of dogs suffering from non-neoplastic diseases, such as, for example, pyometra, were frequently associated with neutrophilia and monocytophilia.

In case of inflammation monocyte-derived macrophages can become reinforcements for the tissue-resident macrophages [18,41].

Macrophages show increased proliferation in the context of inflammatory diseases and, thus, elevated Ki-67 expression, which can lead to higher serum Ki-67 concentrations [42]. Monocytes and lymphocytes, previously mentioned in the context of inflammation, also play an important role in the cancer microenvironment, influencing tumour growth and progression [17,18,20].

To account for inflammation as part of the relationship between tumour tissue and the immune defence mechanisms, we calculated a ratio between the Ki-67 concentrations and the number of immune cells in peripheral blood measured in the accompanying diagnostic routine blood count. This was intended to improve the predictive value of the Ki-67 serum concentration. For example, ratios of lymphocytes to neutrophils were associated with immunohistochemically determined Ki-67 index in the following two recent human medical studies on glioma and on adrenocortical carcinoma, and elevated ratios and elevated Ki-67 indices were associated with higher pathologic grades or increased risk of tumour recurrence [19,21]. To this end, we related and compared serum Ki-67 concentrations and neutrophil, lymphocyte and monocyte counts of mammary adenocarcinoma versus mammary adenoma. However, differentiation between benign and malignant mammary tumours was not possible when using this method in this case. One possible theory for this result would be that the tumour microenvironment was not particularly active at the time of the blood draw or that the corresponding inflammatory cells were more abundant in the surrounding tumour tissue rather than in the peripheral blood.

The immunohistochemical detection of Ki-67 might be superior here [43]. To draw more precise conclusions about this, Ki-67 and optionally inflammatory parameters of interest would have to be measured comparatively in the blood of canine tumour patients and immunohistochemically in the actual tumour tissue in that same study.

In our study, dogs with malignant tumours clearly differed from the comparison groups of healthy dogs and those with non-neoplastic diseases. This fact confirms our hypothesis that Ki-67 is elevated in patients with malignancies and, thus, can be used as a tumour marker. Furthermore, there was no correlation between serum Ki-67 concentration and tumour size. In the literature, tumour size was variably associated with Ki-67 index levels. Although two studies found no correlation with size in pituitary adenomas and soft tissue sarcomas [44,45], others reported Ki-67 expression dependent on tumour size in patients with breast cancer [27].

If Ki-67 elevation in malignancy is independent of tumour size, it may be useful to detect small tumours that may not yet be detectable by imaging modalities. Classical imaging modalities for tumour search reportedly require a minimum mass of 10^9^ cells for visualization [46]. Further studies are needed to elucidate the minimum tumour size required to reliably elevate Ki-67 serum concentrations above the background levels. If used as a screening tool for malignancy, an elevated serum Ki-67 may prompt intensification of tumour search or the use of more sensitive imaging techniques.

In humans with soft tissue sarcomas, a correlation has been found between higher Ki-67 concentrations and poorer prognosis [45,47]. Similarly, in our study some patients with soft tissue sarcomas and high Ki-67 concentrations had short survival times, but these observations were not statistically significant. This might be related to the heterogeneity of the tumours in this group and the small number of cases. In veterinary medicine, different results are found, depending on the tumour type: in hemangiosarcomas of the spleen and mammary adenocarcinomas of the breast, higher Ki-67 indices are associated with a poorer prognosis, whereas in canine apocrine gland anal sac adenocarcinomas no correlation was found regarding survival time [4,48,49].

Lymphomas are a heterogeneous group of tumours with markedly varying but generally high Ki-67 expressions [50]. Not surprisingly, some lymphoma cases in our study had very high serum Ki-67 concentrations.

It should be mentioned that we did not further classify our dogs with malignant lymphoma [51]. We do not expect there to be major differences in serum Ki-67 concentrations between T cell and B cell lymphomas, as high-grade lymphomas usually show a high proliferation rate, independent of immunophenotype. Differences would rather be expected between low-grade and high-grade lymphomas [52].

In immunohistochemistry the Ki-67 expression is mostly evaluated within a respective tumour group, and results can vary here as well. [53].

This restricts the utility of Ki-67 to distinguish between different tumour entities.

The presence of Ki-67 only provides information about the possibility that certain cells might divide, but not about the actual proliferation rate. Therefore, additional tests are recommended, for example another marker [11].

Our results suggest that serum Ki-67 may be useful as a non-selective screening tool for malignancy in health-checks especially in older dogs or in those with increased breed-specific cancer risk [54,55]. Especially in animals presented for a routine check-up lacking history or physical exam findings indicative of inflammation or infection, elevated serum Ki-67 levels should initiate an intensive search for malignant disease using diagnostic imaging and other modalities [56]. The clinical usefulness of any test is based on its sensitivity and specificity [57]. The ROC curve is used in clinical biochemistry to determine the best cut-off for tests and to distinguish between diseased and healthy patients. In addition, the AUC provides an estimate of a test’s usefulness (7). ROC analysis to distinguish between healthy dogs and dogs with malignant tumours revealed that serum Ki-67 concentrations above 1.15 ng/mL were associated with the presence of a malignant tumour with a sensitivity of 0.89 and a specificity of 0.95. Based on our data the AUC was calculated as 0.97. The use of ROC analysis to distinguish between non-neoplastic diseased dogs and dogs with malignant tumours revealed that serum Ki-67 concentrations above 1.9 ng/mL indicate a malignant tumour in the organism, and are associated with a sensitivity of 0.63 and a specificity of 0.62, with an AUC of 0.66. Decreasing the cut-off to 1.26 increases the sensitivity to 0.81 and, thus, the true positive results, but at the same time decreases the specificity to 0.23. Similarly, if the cut-off is set at 2.52, the specificity is increased to 0.81 and the true negative rate is increased, while the sensitivity is decreased to 0.43 [58].

Based on our data, the AUC was calculated as 0.97. Applying the Ki-67-monocyte ratio for ROC analysis to differentiate between non-neoplastic diseased dogs and dogs with malignant tumours improves the AUC up to a number of 0.74 and values above the cut-off 2.15 indicate a malignant tumour in the organism with a sensitivity of 0.71 and a specificity of 0.62.

Some limitations of the present study must be addressed: We did not perform comparative measurements of Ki-67 and inflammatory parameters directly in the tumour tissue of the respective tumour patients, however this would be recommended for future follow-up studies to achieve even more informative results. As long as the direct comparative studies on Ki-67 release from the tumour tissue and the accompanying micro-tumour environment with the corresponding Ki-67 serum levels and inflammatory parameters in the blood are not yet available, we would like to label our results preliminary for the time being. Furthermore, it would be interesting to measure Ki-67 levels again after tumour therapy, and to compare these follow-up levels with the results at the time of diagnosis. To obtain even more conclusive results, follow-up studies with larger numbers of cases, more homogeneous groups, additional benign tumour types, and age-matched control animals would be recommended. This could also help to make a statement about the prognostic value of Ki-67 as a serum tumour marker.

## 5. Conclusions

In summary, Ki-67 may be suitable as a general tumour marker to provide early indication of malignant tumour tissue present in the body during a routine examination, suggesting the need for further diagnostic testing for tumour disease, and it is recommended to also include inflammatory parameters, such as neutrophils, lymphocytes, and monocytes in these examinations.

## Figures and Tables

**Table 1:** Dog breeds of all dogs investigated in this study.

Groups		No.	Dog Breed
Controls		19	Mixed breed (*n* = 6), Small Münsterländer (*n* = 2), Australian Shepherd (*n* = 1), Bodeguero Andaluz (*n* = 1), Border Collie (*n* = 1), Collie (*n* = 1), Dogue de Bordeaux (*n* = 1), German Shepherd (*n* = 1), Golden Doodle (*n* = 1), Irish Wolfhound (*n* = 1), Lagotto Romagnolo (*n* = 1), wire-haired dachshund (*n* = 1), Yorkshire Terrier (*n* = 1)
Non-neoplastic		26	Mixed breed (*n* = 6), Jack Russel Terrier (*n* = 2), Labrador Retriever (*n* = 2), Airedale Terrier (*n* = 1), Austrian Black and Tan Hound (*n* = 1), Beagle (*n* = 1), Bichon Frise (*n* = 1), Curly Coated Retriever (*n* = 1), Dutch Shepherd (*n* = 1), Flat Coated Retriever (*n* = 1), Golden Retriever (*n* = 1), Greater Swiss Mountain Dog (*n* = 1), long-haired dachshund (*n* = 1), Plott Hound (*n* = 1), Ratonero Mallorquín (*n* = 1), Sealyham Terrier (*n* = 1), Small Münsterländer (*n* = 1), West Highland White Terrier (*n* = 1), White Swiss Shepherd Dog (*n* = 1)
Malignant Tumours	Carcinoma	35	Mixed breed (*n* = 7), Labrador Retriever (*n* = 3), Bavarian Mountain Hound (*n* = 2), Beagle (*n* = 2), Collie (*n* = 2), German Shepherd (*n* = 2), Boxer (*n* = 1), Cocker Spaniel (*n* = 1), Entlebucher Mountain Dog (*n* = 1), Golden Retriever (*n* = 1), Harz fox (*n* = 1), Havanese (*n* = 1), Hovawart (*n* = 1), Magyar Vizsla (*n* = 1), Miniature Schnauzer (*n* = 1), Old German shepherd dog (*n* = 1), Pitbull (*n* = 1), Rhodesian Ridgeback (*n* = 1), Samoyed dog (*n* = 1), Schnauzer (*n* = 1), Shetland Sheepdog (*n* = 1), Welsh Terrier (*n* = 1), wire-haired dachshund (*n* = 1)
	Mesenchymal tumours	26	Mixed breed (*n* = 9), Jack Russel Terrier (*n* = 3), Labrador Retriever (*n* = 3), Bernese Mountain Dog (*n* = 2), German Shepherd (*n* = 2), Australian Shepherd (*n* = 1), Beagle (*n* = 1), Boxer (*n* = 1), Golden Retriever (*n* = 1), Kooikerhondje (*n* = 1), Podenco (*n* = 1), Rottweiler (*n* = 1)
	Lymphoma	21	Mixed breed (*n* = 8), Alaskan Malamute (*n* = 1), Cocker Spaniel (*n* = 1), Dandie Dinmont Terrier (*n* = 1), Galgo Español (*n* = 1), German Shepherd (*n* = 1), Golden Retriever (*n* = 1), Gordon Setter (*n* = 1), Great Dane (*n* = 1), Irish Terrier (*n* = 1), Jack Russel Terrier (*n* = 1), Miniature Schnauzer (*n* = 1), Rhodesian Ridgeback (*n* = 1), Rottweiler (*n* = 1)
Benign Tumours	Mammary Adenoma	13	Rhodesian Ridgeback (*n* = 2), Yorkshire Terrier (*n* = 2), Australian Shepherd (*n* = 1), Bichon Frise (*n* = 1), German Shepherd (*n* = 1), German Wirehaired Pointer (*n* = 1), Golden Retriever (*n* = 1), Hovawart (*n* = 1), Miniature Bull Terrier (*n* = 1), Pomeranian (*n* = 1), Russian Toy (*n* = 1)

**Table 2:** Number, sex, and age of all dogs investigated in this study.

Groups		No. (f/m)	Age (yr) Min	Max	Mean
Controls		19 (9/10)	0.5	10	5.0
Non-neoplastic		26 (12/14)	1.3	17.3	8.4
Malignant tumours	Total	82 (57/25)	4.5	16	10.1
Carcinoma (1)	Mammary Adenocarcinoma	26 (26/0)	6.5	15.5	11
Carcinoma (2)	Squamous cell carcinoma	9 (5/4)	6	16	10.5
Mesenchymal tumours (1)	Subcutaneous spindle cell sarcoma	17 (9/8)	5	14	9.8
Mesenchymal tumours (2)	Splenic hemangiosarcoma	9 (4/5)	5	15	10.3
	Lymphoma	21 (13/8)	4.5	14	9.2
Benign tumours	Mammary Adenoma	13 (13/0)	6	12	9.3

**Table 3:** Tumour volume.

Groups		Min (cm^3^)	Max (cm^3^)	Mean (cm^3^)	Median (cm^3^)
Malignant Tumours					
Carcinoma (1)	Mammary Adenocarcinoma	0.07	321.0	42.79	14.0
Carcinoma (2)	Squamous cell carcinoma	1.7	86.5	26.6	8.1
Mesenchymal tumours (1)	Subcutaneous spindle cell sarcoma	0.26	465.92	100.87	40.87
Mesenchymal tumours (2)	Splenic hemangiosarcoma	1.17	399.36	147.86	134.55
Benign Tumours					
	Mammary Adenoma	0.1	22.4	2.38	0.5

**Table 4:** Neutrophils, lymphocytes, and monocytes of dogs in this study.

Groups		Min (K/μL)	Max (K/μL)	Mean (K/μL)	Median (K/μL)
Controls	Neutrophils	3.24	10.27	6.07	6.51
	Lymphocytes	1.06	4.1	2.17	2.1
	Monocytes	0.25	1.18	0.58	0.49
Non-neoplastic	Neutrophils	3.57	31.55	10.87	7.19
	Lymphocytes	0.77	5.6	2.5	2.17
	Monocytes	0.3	4.07	1.3	1.0
Malignant Tumours					
Carcinoma (1): Mammary Adenocarcinoma	Neutrophils	3.05	27.66	7.92	5.79
	Lymphocytes	0.77	4.73	1.80	1.72
	Monocytes	0.28	3.61	0.72	0.58
Carcinoma (2): Squamous cell carcinoma	Neutrophils	5.0	10.99	7.2	6.56
	Lymphocytes	1.56	2.59	1.84	1.67
	Monocytes	0.4	1.24	0.71	0.58
Mesenchymal tumours (1): Subcutaneous spindle cell sarcoma	Neutrophils	3.75	19.54	6.67	5.92
	Lymphocytes	0.8	2.63	1.86	1.97
	Monocytes	0.13	2.87	0.65	0.5
Mesenchymal tumours (2): Splenic hemangiosarcoma	Neutrophils	3.9	19.56	11.0	10.94
	Lymphocytes	0.58	7.63	2.17	1.62
	Monocytes	0.1	5.66	1.65	1.25
Lymphoma	Neutrophils	0.57	38.66	7.76	5.19
	Lymphocytes	0.43	7.96	2.02	1.44
	Monocytes	0.19	4.18	1.17	0.68
Benign tumours					
Mammary Adenoma	Neutrophils	0.34	15.53	7.35	7.18
	Lymphocytes	1.06	4.49	2.15	1.9
	Monocytes	0.36	4.15	0.98	0.58

**Table 5:** Ratios of Ki-67 and neutrophil, lymphocyte and monocyte counts of all dogs examined in this study.

Groups		Min	Max	Mean	Median
Controls	Ki-67-neutrophil-ratio	0.0	0.57	0.09	0.06
	Ki-67-lymphocyte-ratio	0.0	1.01	0.25	0.13
	Ki-67-monocyte-ratio	0.0	7.52	1.16	0.58
Non-neoplastic	Ki-67-neutrophil-ratio	0.03	0.71	0.23	0.17
	Ki-67-lymphocyte-ratio	0.16	1.96	0.89	0.82
	Ki-67-monocyte-ratio	0.44	6.12	2.12	1.44
Malignant Tumours					
Carcinoma (1): Mammary Adenocarcinoma	Ki-67-neutrophil-ratio	0.05	4.60	0.62	0.39
	Ki-67-lymphocyte-ratio	0.31	7.46	2.13	1.59
	Ki-67-monocyte-ratio	1.05	50.14	6.91	4.09
Carcinoma (2): Squamous cell carcinoma	Ki-67-neutrophil-ratio	0.09	2.92	0.9	0.37
	Ki-67-lymphocyte-ratio	0.45	8.11	2.57	1.49
	Ki-67-monocyte-ratio	0.86	34.08	8.73	4.99
Mesenchymal tumours (1): Subcutaneous spindle cell sarcoma	Ki-67-neutrophil-ratio	0.08	1.71	0.52	0.37
	Ki-67-lymphocyte-ratio	0.45	6.21	1.65	1.09
	Ki-67-monocyte-ratio	0.54	16.54	6.94	7.61
Mesenchymal tumours (2): Splenic hemangiosarcoma	Ki-67-neutrophil-ratio	0.1	0.66	0.33	0.26
	Ki-67-lymphocyte-ratio	0.61	4.53	2.0	1.55
	Ki-67-monocyte-ratio	0.66	9.9	3.85	3.42
Lymphoma	Ki-67-neutrophil-ratio	0.03	29.23	2.15	0.34
	Ki-67-lymphocyte-ratio	0.13	20.83	3.42	1.45
	Ki-67-monocyte-ratio	0.30	84.61	7.84	2.91
Benign Tumours					
Mammary Adenoma	Ki-67-neutrophil-ratio	0.11	5.68	0.89	0.35
	Ki-67-lymphocyte-ratio	0.42	4.11	1.61	1.21
	Ki-67-monocyte-ratio	0.47	9.67	4.29	3.96

## Data Availability

The data presented in this study are available upon request from the corresponding author. The data are not publicly accessible due to data protection.
